# Genetic Polymorphisms of the *TYMS* Gene Are Not Associated with Congenital Cardiac Septal Defects in a Han Chinese Population

**DOI:** 10.1371/journal.pone.0031644

**Published:** 2012-02-23

**Authors:** Jian-Yuan Zhao, Jing-Wei Sun, Zhuo-Ya Gu, Jue Wang, Er-Li Wang, Xue-Yan Yang, Bin Qiao, Wen-Yuan Duan, Guo-Ying Huang, Hong-Yan Wang

**Affiliations:** 1 The State Key Laboratory of Genetic Engineering and MOE Key Laboratory of Contemporary Anthropology, School of Life Sciences, Fudan University, Shanghai, China; 2 Institute of Cardiovascular Disease General Hospital, Jinan Military Region, Jinan, China; 3 Children's Hospital Shanghai, Fudan University, Shanghai, China; 4 The Institutes of Biomedical Sciences, Fudan University, Shanghai, China; 5 Institute of Sports Science and Technology, Administration of Sports of Anhui Province, Hefei, China; Brigham and Women's Hospital and Harvard Medical School, United States of America

## Abstract

**Background:**

Clinical research indicates that periconceptional administration of folic acid can reduce the occurrence of congenital cardiac septal defects (CCSDs). The vital roles of folate exhibits in three ways: the unique methyl donor for DNA expression regulation, the *de novo* biosynthesis of purine and pyrimidine for DNA construction, and the serum homocysteine removal. Thymidylate synthase (*TYMS*) is the solo catalysis enzyme for the *de novo* synthesis of dTMP, which is the essential precursor of DNA biosynthesis and repair process. To examine the role of *TYMS* in Congenital Cardiac Septal Defects (CCSDs) risk, we investigated whether genetic polymorphisms in the *TYMS* gene associated with the CCSDs in a Han Chinese population.

**Method:**

Polymorphisms in the noncoding region of *TYMS* were identified via direct sequencing in 32 unrelated individuals composed of half CCSDs and half control subjects. Nine SNPs and two insertion/deletion polymorphisms were genotyped from two independent case-control studies involving a total of 529 CCSDs patients and 876 healthy control participants. The associations were examined by both single polymorphism and haplotype tests using logistic regression.

**Result:**

We found that *TYMS* polymorphisms were not related to the altered CCSDs risk, and even to the changed risk of VSDs subgroup, when tested in both studied groups separately or in combination. In the haplotype analysis, there were no haplotypes significantly associated with risks for CCSDs either.

**Conclusion:**

Our results show no association between common genetic polymorphisms of the regulatory region of the *TYMS* gene and CCSDs in the Han Chinese population.

## Introduction

Congenital heart disease is one of the most common birth defects and among the leading causes of infant death worldwide, with an incidence of 19 to 75 per 1,000 live births [1]. Clinical research over the past twenty years suggests that maternal periconceptional folic acid supplementation would reduce the occurrence of congenital heart disease by 40–60%, especially for congenital cardiac septal defects (CCSDs) and conotruncal defects [2]–[9]. Moreover, folate antagonists increase congenital heart disease risk, particularly CCSDs and conotruncal malformations [10].

Folate acts as a one-carbon donor, which is involved in both the *de novo* synthesis of nucleotides and methyl transfer reactions. Therefore exploring the association between genetic variants in genes involved in folate metabolism pathway and the risk of CCSD will shed light on the mechanism how folate carries out its protection effects. Considering the interacted environmental factors, it was speculated that fetuses with the genetic susceptibility will be more fragile when challenged by the maternal absence of folate, especially during the embryonic heart development period [11]–[13]. So folate supplement should be supplemented during the first trimester of pregnancy.

Thymidylate synthase (*TYMS*) is the key enzyme in the *de novo* synthesis of 2′-deoxyuridine -5′-monophosphate (dTMP), which is the essential precursor of DNA biosynthesis and repair process [14]. Numerous studies have shown that the polymorphisms in *TYMS* gene influence the enzyme's activity and correlate with the folate levels [15]–[18]. The most extensively reported *TYMS* variants are two insertion/deletion sites in 5′UTR (rs34743033) and 3′UTR (rs34489327), respectively, which are associated with the occurrence of various tumors, such as colorectal cancer, lymphoma, and acute lymphocytic leukemia [16], [19], [20].

Although the *TYMS* plays an important role in the developing fetus and is closely related to many diseases, so far it has not yet been reported to be associated with CCSDs in Han Chinese population. In this study, we investigated the effects of two extensively studied *TYMS* insertion/deletion sites on CCSDs, including ventricular septal defects (VSDs), atrial septal defects (ASDs) and complex traits composed of VSD and ASD, in a Han Chinese population. Moreover, we extended this association study to additional 9 common SNP sites (MAF>0.1) by sequencing and analyzing the promoter, 5′UTR and 3′UTR regulatory regions of *TYMS*.

## Materials and Methods

### Study subjects

We analyzed samples from two independent case-control groups. The Shanghai group consisted of 270 CCSDs patients and 552 matched healthy controls. These patients and controls were enrolled between August 2008 and February 2011 from the Children's Hospital of Fudan University (Shanghai, China). The Shandong group consisted of 259 CCSDs patients and 324 healthy controls. These patients and controls were recruited between August 2008 and January 2011 from the Cardiovascular Disease Institute, General Hospital of Jinan Military Command (Jinan, Shandong Province, China). Among the 529 CCSDs patients, 447 had VSD, 31 ASD, 37 complex traits composed of VSD and ASD and 14 other complex traits involved VSD. All of the controls were non-CCSDs out-patients from the same geographic area who were matched to affected individuals by age and sex during the same period (Table 1). All subjects were genetically unrelated ethnic Han Chinese. CCSDs patients who had structural malformations involving another organ system or positive family history of CCSDs in first-degree relatives (parents, siblings and children) were excluded.

**Table 1:** Demographic characteristics in CCSDs cases and controls.

Variable	Cases		Controls		P value*
	No.	%	No.	%	
Shanghai Group	N = 270		N = 552		
Age, years (mean±SEM)	4.46±0.43		4.39±0.15		0.87
Gender					0.81
Male	160	59.3	332	60.1	
Female	110	40.7	220	39.9	
Shandong Group	N = 259		N = 324		
Age, years (mean±SEM)	7.93±0.32		8.07±0.10		0.68
Gender					0.96
Male	118	45.6	147	45.3	
Female	141	54.4	177	54.6	
Combined Group	N = 529		N = 876		
Age, years (mean±SEM)	6.16±0.28		5.75±0.12		0.17
Gender					0.44
Male	278	52.6	479	54.7	
Female	251	47.4	397	45.3	

*All comparisons by 2-side χ2 test. Date shown in the table is means (±SEM).

To screen non-coding variants in the *TYMS* gene, 32 unrelated individuals consisting of 16 CCSDs patients and 16 controls from the Shanghai and Shandong groups were randomly selected for resequencing. All study protocols were reviewed and approved by the ethics committee of School of Life Science, Fudan University, and written consents were obtained from parents and/or patients prior to commencing the study.

### SNP identification and genotyping

Genomic DNA was isolated from venous blood using conventional regents. The *TYMS* non-coding region from −2132 to +447 bp (2579 bp, chr18: 645519–648098, NC_000018.8, GI: 51511735) and the fragment containing the whole 3′UTR (742 bp, chr18: 662863–663605, NC_000018.8, GI: 51511735) were amplified by PCR from 32 unrelated individuals randomly selected from both the Shanghai and Shandong groups for variant screening using sequencing. Direct dye terminator sequencing of PCR products was performed using the ABI Prism BigDye system according to the manufacturer's instructions (ABI, Foster City, CA, USA). Selected SNPs were genotyped using SNAPshot analysis (ABI, Foster City, CA, USA). Selected insertion/deletion sites were genotyped using multi-PCR amplification. Sequencing and genotyping samples were processed on an ABI 3730 automated sequencer and analyzed using SeqMan and Peakscan, respectively. All DNA sequences of primer pairs were listed in Table S1.

### Statistical analysis

Differences in demographic features, allelic or genotypic frequencies between cases and controls were compared using the χ^2^ test. The Hardy-Weinberg equilibrium was also tested by a χ^2^ test in the controls. To evaluate the associations between genotypes and CCSDs risk, the odds ratios (ORs) and 95% confidence intervals (CIs) were calculated by unconditional logistic regression analysis with adjustment for age and sex.

The estimation of the haplotype frequency and the analysis of associations between different haplotypes and CCSDs risk were performed by the SNPStats web tool (http://bioinfo.iconcologia.net/snpstats/start.htm) with adjustment for age and sex [21].

## Results

By resequencing the 32 randomly chosen samples in the non-coding region of *TYMS*, 19 polymorphisms were identified, including 15 SNPs and 4 insertion/deletion polymorphisms. Among the 19 polymorphisms, 10 SNPs and 3 insertion/deletion polymorphisms were found in dbSNP of the NCBI database and 5 SNPs and 1 insertion/deletion polymorphism were novel. According to the criteria for common variants, we chose 9 SNP and 2 insertion/deletion polymorphisms with MAF>0.1 for further exploration in both cohorts, which were rs58808873, rs9967368, rs56697663, rs2853741, rs2606241, rs9952504, rs34743033, rs73366471, rs699517, rs2790 and rs34489327.

We genotyped all 11 polymorphisms in 270 cases versus the 552 controls in the Shanghai group and 259 cases versus the 324 controls in the Shandong group. All genotype frequencies were in accordance with the Hardy-Weinberg equilibrium among control subjects (*P*>0.05). The allelic and genotypic frequencies of the 11 polymorphisms are listed in Table 2 and Table S2. The minor allele frequency (MAF) for each polymorphism in our case/control subjects was consistent with published data of Han Chinese population in dbSNP database. In the association study, we found that the genotypic distribution of all studied polymorphisms were not significantly different between the CCSDs patients and control subjects in both two case/control groups and the combined dataset. A stratified analysis was also performed in VSD subgroup, as shown in Table S3, we did not observe any associations between studied polymorphisms and VSD. These results indicate that these polymorphisms are not significantly associated with the occurrence of congenital cardiac septal defects, including the two most extensively reported insertion/deletion sites, rs34743033 and rs34489327.

**Table 2:** The allelic and genotype frequency of the *TYMS* polymorphisms in CCSD patients and controls.

SNP ID	Chromosome Position	Base change	Location	Group		MAF		GenotypeP$	HWEP&
					Case	Control	Database*		
rs58808873	645876	C>T	Promoter region	Shanghai	0.174	0.175	0.200	0.65	0.37
				Shandong	0.192	0.164		0.28	0.31
rs9967368	646021	C>G	Promoter region	Shanghai	0.432	0.442	0.427	0.80	0.55
				Shandong	0.462	0.442		0.70	0.18
rs56697663	647278	−>C	Promoter region	Shanghai	0.391	0.393	N.A.#	0.91	0.72
				Shandong	0.421	0.408		0.81	0.25
rs2853741	647353	T>C	Promoter region	Shanghai	0.462	0.499	0.475	0.31	0.27
				Shandong	0.491	0.485		0.92	0.12
rs2606241	647444	A>C	Promoter region	Shanghai	0.371	0.347	0.425	0.27	1
				Shandong	0.408	0.431		0.50	0.11
rs9952504	647459	A>G	Promoter region	Shanghai	0.0760	0.0695	0.092	0.90	0.17
				Shandong	0.106	0.0890		0.61	0.30
rs34743033	647675	I>D	5′UTR	Shanghai	0.202	0.201	0.237	0.53	0.43
				Shandong	0.195	0.204		0.89	0.49
rs73366471	648036	A>G	Intron 1	Shanghai	0.0740	0.0660	N.A.#	0.80	0.29
				Shandong	0.0545	0.0555		0.96	0.25
rs699517	663017	T>C	3′UTR	Shanghai	0.313	0.285	0.300	0.49	1
				Shandong	0.301	0.345		0.28	0.39
rs2790	663087	A>G	3′UTR	Shanghai	0.368	0.390	0.375	0.22	0.089
				Shandong	0.423	0.389		0.17	0.91
rs34489327	663445	D>I	3′UTR	Shanghai	0.311	0.316	0.302	0.71	0.17
				Shandong	0.333	0.326		0.97	0.38

*MAF, minor allele frequency from HapMap database for CHB population.

$ P value for difference in genotypes distributions between case and control subjects.

& P value for Hardy-Weinberg equilibrium test in the control subjects. Additional detailed genotype frequencies is present in Table S2.

# Not available in dbSNP database.

In the haplotypic analysis, we reconstructed the haplotype for the whole gene based on studied polymorphisms to assess risks for both the case-control groups and the combined group. As shown in Table S4, there were two haplotypes increasing the risk of CCSDs in the Shanghai group, but the frequencies of these haplotypes were very low (Frequency of No. 11 haplotype = 0.0163, P = 0.04; Frequency of No. 14 haplotype = 0.0136, P = 0.04). Moreover, these two haplotypes didn't exist in Shandong group where we hadn't observed any other associations between haplotypes and CCSDs risk (Table S5). To exclude the possibility that the different haplotype analysis results from two cohorts were due to the hidden population stratification, we tested the shared high frequency haplotypes (Frequency>5%) distributions in control groups and all sample set of both cohorts, respectively. The results showed that there was no population substructure existed (Table S6). Therefore, we performed the haplotype analysis In the combined group and found that there were no haplotypes significantly associated with risks for CCSDs (Table 3). These two positive haplotypes in Shanghai group might attribute to the bias from limited sample size and low frequency. Further study will be warranted with the enlarged sample size for a conclusive results.

**Table 3:** *TYMS* haplotype analysis of combined group.

No.	rs58808873	rs9967368	rs56697663	rs2853741	rs2606241	rs9952504	rs34743033	rs73366471	rs699517	rs2790	rs34489327	Freq(Case)	Freq(Control)	Freq(Total)	OR (95% CI)*	P-value$
1	G	C	T	A	A	T	I	A	T	C	D	0.1647	0.1912	0.1757	1.00	---
2	G	C	T	A	A	T	I	A	T	T	D	0.1055	0.1125	0.107	1.20(0.84–1.73)	0.32
3	G	G	T	A	A	T	I	A	T	C	D	0.0709	0.0621	0.0648	1.42(0.92–2.17)	0.11
4	A	G	C	G	C	T	I	A	C	T	I	0.0571	0.0584	0.0564	1.20(0.78–1.84)	0.4
5	G	G	C	G	C	T	D	A	C	T	I	0.0329	0.0313	0.0317	1.27(0.74–2.19)	0.39
6	G	C	C	G	C	T	I	A	T	T	D	0.0312	0.0281	0.029	0.95(0.51–1.80)	0.89
7	G	G	C	G	C	T	I	A	C	T	I	0.0246	0.0297	0.0287	1.36(0.78–2.38)	0.28
8	G	C	C	G	C	T	D	A	C	T	I	0.0285	0.0261	0.0272	1.24(0.68–2.25)	0.48
9	A	G	C	G	C	T	D	A	C	T	I	0.0222	0.0268	0.0263	1.02(0.57–1.83)	0.94
10	G	C	T	A	A	T	D	A	T	C	D	0.017	0.0203	0.0208	1.10(0.51–2.41)	0.8
11	G	G	C	G	C	T	I	A	T	T	D	0.0183	0.0125	0.0158	1.74(0.79–3.86)	0.17
12	G	C	T	A	C	T	I	A	T	C	D	0.0162	0.0126	0.0151	1.63(0.80–3.31)	0.18
13	G	C	C	G	C	T	I	A	C	T	I	0.0149	0.0124	0.0147	1.24(0.53–2.89)	0.61
14	G	C	T	G	A	T	I	A	T	C	D	0.0144	0.0103	0.013	0.80(0.34–1.89)	0.6
15	G	C	C	G	A	T	I	A	T	T	D	0.0156	0.011	0.0129	1.59(0.67–3.78)	0.3
16	G	C	T	A	A	T	D	A	T	T	D	0.0115	0.0129	0.0129	1.64(0.64–4.21)	0.3
17	A	G	C	G	C	T	I	A	T	C	D	0.014	0.01	0.0124	1.70(0.71–4.04)	0.23
18	G	G	C	G	A	T	D	A	C	T	I	0.0079	0.0135	0.0115	0.66(0.24–1.85)	0.43
19	G	G	T	G	C	C	I	G	T	T	D	0.0115	0.0101	0.0106	1.49(0.62–3.57)	0.37
20	G	C	T	A	A	C	I	A	T	C	D	0.0081	0.0106	0.0102	1.21(0.40–3.63)	0.74
Rare#	*	*	*	*	*	*	*	*	*	*	*	0.313	0.2976	0.3036	1.29(0.98–1.71)	0.072

*Adjusted by age and gender;

$ P value for difference in haplotypes distributions between case and control subjects;

# The sum of other rare haplotypes.

## Discussion

Epidemiological evidence indicates that periconceptional administration of folic acid leads to a reduction of the risk of delivering newborns with congenital heart disease, especially in cardiac septal defects and conotruncal cardiac defects, which makes exploring the association between genetic variants in folate-related genes and the risk of congenital heart disease an attractive pursuit. *TYMS* is the exclusive gene for *de novo* synthesis of dTMP, which is essential for DNA synthesis. Many studies were performed to investigate the relation between mutations/polymorphisms of coding region of *TYMS* and various diseases, such as colorectal cancer, lymphoma and acute lymphocytic leukemia. Polymorphisms in the noncoding region, however, have been largely ignored except for two extensively studied insertion/deletion polymorphisms, and limited studies have been conducted in congenital heart disease, which is affected by the folate level directly.

In the current study, we explored the relationship between polymorphisms in the non-coding region (including promoter region, 5′UTR and 3′UTR) of *TYMS* and congenital cardiac septal defects, and 11 common polymorphisms were selected for genotyping in the association study of two independent groups. As a consequence, none of specific disease-related polymorphisms or haplotypes have been found, which indicate that the polymorphic *TYMS'*s non-coding region is not associated with the elevated cardiac septal defects risk in the Han Chinese population. Other studies performed in conotruncal cardiac defects obtained similar results although different regulatory polymorphisms and ethnic groups were used in association studies [9], [15]. Taking into account all the investigations above, no sufficient evidence has been demonstrated for a significant association between *TYMS* polymorphisms and congenital heart disease,which suggests that the protection effects of folate administration for CCSDs may not be directly influenced by the pyrimidine synthesis.

The folate-dependent TYMS shares the same substrate (5,10-methylene tetrahydrofolate, methylene-5THF) with 5,10-methylenetetrahydrofolate reductase (MTHFR). Consequently, the available TYMS for folate is competitively affected by MTHFR. It was previously described that because the recycle from methylene-5THF to the folate pool is catalyzed mainly by MTHFR, the controls of the plasma folate concentration were significantly attributed to the *MTHFR* polymorphisms compared with *TYMS* polymorphisms [16]. Here, our results support that the *TYMS* polymorphisms do not perform as an independent genetic risk factor for the elevated incidence of congenital cardiac septal defects. Additionally, the pathway of folate metabolism is in charge of de novo DNA precursor synthesis and methylation [22]. The occurrence of CCSDs might be attributed more to the methylation, considering DNA or protein methylation would play a significant role in the embryo heart development. The negative results derived from present study could be alternatively explained by the central role *TYMS* played in DNA synthesis and repair. The *TYMS* mutations leading to function or dosage mutation may create a powerful selective effect by resulting in great detriments on embryonic development, such as serious defects or even death. However, the samples of CCSDs examined in this study, characterized as a type of medium and mild congenital heart disease, cannot tolerate severe functional mutations. This hypothesis could be corroborated by the results of the exon's sequencing of *TYMS* in the same examined samples, where no case of missense mutation has been found in the *TYMS* entire coding region of 200 ventricular septal defect samples (data not shown). Moreover, a potential limitation might also be imposed on the presence of polymorphisms with severe regulatory malfunctions in the non-coding region.

With respect to the characteristics of *TYMS*'s structure, no canonical eukaryotic transcriptional signals such as the TATA box, CAAT box and GC box are found in the 5′ upstream region of *TYMS*. Instead, three sequences consistent with GC boxes were reported by Kaneda in intron-1 [23] and other regulatory sequences were further identified within intron-1, which could stimulate the *TYMS* gene expression in concert with the 5′ non-coding region [24]. Furthermore, many common polymorphic sites were identified in the *TYMS* promoter and UTR region in the current study, indicating a high tolerance for polymorphisms (one SNP per 160 bp). Consequently, we postulate that the promoter and UTR region may not be the core regulatory region for the expression of *TYMS* gene, while intron-1 of *TYMS* might play a more associative role in the regulation of gene expression [25]. In addition, it is worth mentioning that according to a previous study, the regulation of *TYMS* mRNA level may attribute mostly to post-transcriptional events in humans [26]. Thus, more attention should be placed on intron-1 in the future research related to *TYMS* expression and function.

In addition, our study is limited to the neonatal genotype and lacks research for the maternal genotype. It is also possible that the protective effect of periconceptional supplement of folic acid is generated by the adjustment of the maternal metabolic defect, which is associated closely with maternal genotype instead of the neonatal genotype. The information of maternal dietary folate intake during pregnancy is also not available in this study. It was noted that the strongest associations between polymorphisms of folate-related genes and congenital heart defects might be observed only when environmental factors are taken into consideration [11]–[13]. We can't exclude the possibility that the effect of *TYMS* polymorphisms might be influenced by the interacted maternal folate status. Additional studies will be required to determine the levels of maternal serum folate concentration, which would help to figure out valuable polymorphisms in *TYMS*.

## Supporting Information

Table S1 **DNA sequence of all used primer pairs.**
(DOC)

Table S2 **The genotype frequency of the 11 identified TYMS polymorphisms in CCSDs patients and controls.**
(DOC)

Table S3 **Associations between** ***TYMS*** **polymorphisms and VSD in two independent case-control studies.**
(DOC)

Table S4 ***TYMS*** **main haplotype (Frequence>0.01) analysis of Shanghai group.**
(DOC)

Table S5 ***TYMS*** **main haplotype (Frequence>0.01) analysis of Shandong group.**
(DOC)

Table S6 **High frequency haplotypes (Frequency>5%) distribution in Shanghai and Shandong group.**
(DOC)
