# Supplementary material for: Genetic Polymorphisms of the TYMS Gene Are Not Associated with Congenital Cardiac Septal Defects in a Han Chinese Population
Source: PLoS One. 2012 Feb 23;7(2):e31644. doi: 10.1371/journal.pone.0031644 (PMC3285645; doi:10.1371/journal.pone.0031644)
Supplement: Table S1 — DNA sequence of all used primer pairs. (DOC) [file pone.0031644.s001.doc]

Table S1.DNA sequence of all used primer pairs

| PRIMER NAME | SEQUENCE (5’-3’) | PURPOSE |
| --- | --- | --- |
| TYMS-Seq-1F | TCCTGCTAAAGCCCTGCAAT | PCR/SEQUENCE |
| TYMS-Seq-1R | GCGGAGGTTGCTTGCAGTAG | PCR/SEQUENCE |
| TYMS-Seq-2F | GAATCCATGGTCTCCACAACC | PCR/SEQUENCE |
| TYMS-Seq-2R | TCGGCCCCAAGTTTTTAATAGA | PCR/SEQUENCE |
| TYMS-Seq-3F | GGGTTTCACCATGTTGTCCA | PCR/SEQUENCE |
| TYMS-Seq-3R | GGGCCACAGCTGAGAGTCTT | PCR/SEQUENCE |
| TYMS-Seq-4F | TCAGCGAGAACCCAGACCTT | PCR/SEQUENCE |
| TYMS-Seq-4R | GCATCCAAACCAGAATACAGCA | PCR/SEQUENCE |
| rs34743033typing-F | cctggcgcacgctctcta | In/del-test |
| rs34743033typing-R | gcggaggatgtgttggatct | In/del-test |
| rs34489327typing-F | tgtgcatttcaatcccacgta | In/del-test |
| rs34489327typing-R | gaacaaagcgtggacgaatg | In/del-test |
| rs58808873typing | ATTGACATCAACACAGGCCT | SNaPshot |
| rs9967368typing | CCCAGATATTCCTTTCTATT | SNaPshot |
| rs56697663typing | ATACCACTTGCTTCGGTTGC | SNaPshot |
| rs2853741typing | ATCTCAAACAGCAGTTTTGT | SNaPshot |
| rs2606241typing | CAAAGGGCGCAGTCCTTCCC | SNaPshot |
| rs9952504typing | TTCCCAGCCACCGCACCTGC | SNaPshot |
| rs73366471typing | ggagctgccgggcgctgcgg | SNaPshot |
| rs699517typing | AGGGTGCTTTCAAAGGAGCT | SNaPshot |
| rs2790typing | GATTTTTACCTAGTTCCTT | SNaPshot |

Table S2. The genotype frequency of the 11 identified *TYMS* polymorphisms in CCSDs patients and controls

| SNP | Group | Genotype | Case | Control |
| --- | --- | --- | --- | --- |
| rs58808873 | Shanghai | C/C | 185 (68.5%) | 373 (67.6%) |
|  |  | C/T | 76 (28.1%) | 166 (30.1%) |
|  |  | T/T | 9 (3.3%) | 13 (2.4%) |
|  | Shandong | C/C | 176 (68%) | 229 (70.7%) |
|  |  | C/T | 67 (25.9%) | 84 (25.9%) |
|  |  | T/T | 16 (6.2%) | 11 (3.4%) |
| rs9967368 | Shanghai | C/C | 88 (32.6%) | 168 (30.4%) |
|  |  | G/C | 131 (48.5%) | 280 (50.7%) |
|  |  | G/G | 51 (18.9%) | 104 (18.8%) |
|  | Shandong | C/C | 77 (29.7%) | 107 (33%) |
|  |  | G/C | 125 (48.3%) | 148 (45.7%) |
|  |  | G/G | 57 (22%) | 69 (21.3%) |
| rs56697663 | Shanghai | -/- | 101 (37.4%) | 201 (36.4%) |
|  |  | -/C | 127 (47%) | 268 (48.5%) |
|  |  | C/C | 42 (15.6%) | 83 (15%) |
|  | Shandong | -/- | 89 (34.4%) | 119 (36.7%) |
|  |  | -/C | 122 (47.1%) | 146 (45.1%) |
|  |  | C/C | 48 (18.5%) | 59 (18.2%) |
| rs2853741 | Shanghai | T/T | 73 (27%) | 132 (23.9%) |
|  |  | T/C | 145 (53.7%) | 290 (52.5%) |
|  |  | C/C | 52 (19.3%) | 130 (23.6%) |
|  | Shandong | T/T | 71 (27.4%) | 93 (28.7%) |
|  |  | T/C | 122 (47.1%) | 148 (45.7%) |
|  |  | C/C | 66 (25.5%) | 83 (25.6%) |
| rs2606241 | Shanghai | A/A | 101 (37.4%) | 236 (42.8%) |
|  |  | C/A | 138 (51.1%) | 250 (45.3%) |
|  |  | C/C | 31 (11.5%) | 66 (12%) |
|  | Shandong | A/A | 101 (39%) | 112 (34.6%) |
|  |  | C/A | 105 (40.5%) | 145 (44.8%) |
|  |  | C/C | 53 (20.5%) | 67 (20.7%) |
| rs9952504 | Shanghai | A/A | 232 (85.9%) | 480 (87%) |
|  |  | A/G | 35 (13%) | 67 (12.1%) |
|  |  | G/G | 3 (1.1%) | 5 (0.9%) |
|  | Shandong | A/A | 208 (80.3%) | 270 (83.3%) |
|  |  | A/G | 47 (18.1%) | 50 (15.4%) |
|  |  | G/G | 4 (1.5%) | 4 (1.2%) |
| rs34743033 | Shanghai | I/I | 174 (64.4%) | 349 (63.2%) |
|  |  | I/D | 83 (30.7%) | 184 (33.3%) |
|  |  | D/D | 13 (4.8%) | 19 (3.4%) |
|  | Shandong | I/I | 165 (63.7%) | 203 (62.6%) |
|  |  | I/D | 87 (33.6%) | 110 (34%) |
|  |  | D/D | 7 (2.7%) | 11 (3.4%) |
| rs73366471 | Shanghai | A/A | 233 (86.3%) | 483 (87.5%) |
|  |  | A/G | 34 (12.6%) | 65 (11.8%) |
|  |  | G/G | 3 (1.1%) | 4 (0.7%) |
|  | Shandong | A/A | 233 (90%) | 290 (89.5%) |
|  |  | A/G | 24 (9.3%) | 32 (9.9%) |
|  |  | G/G | 2 (0.8%) | 2 (0.6%) |
| rs699517 | Shanghai | T/T | 128 (47.4%) | 282 (51.1%) |
|  |  | C/T | 115 (42.6%) | 226 (40.9%) |
|  |  | C/C | 27 (10%) | 44 (8%) |
|  | Shandong | T/T | 131 (50.6%) | 143 (44.1%) |
|  |  | C/T | 100 (38.6%) | 139 (42.9%) |
|  |  | C/C | 28 (10.8%) | 42 (13%) |
| rs2790 | Shanghai | A/A | 110 (40.7%) | 196 (35.5%) |
|  |  | A/G | 121 (44.8%) | 282 (51.1%) |
|  |  | G/G | 39 (14.4%) | 74 (13.4%) |
|  | Shandong | A/A | 78 (30.1%) | 120 (37%) |
|  |  | A/G | 143 (55.2%) | 156 (48.1%) |
|  |  | G/G | 38 (14.7%) | 48 (14.8%) |
| rs34489327 | Shanghai | D/D | 128 (47.4%) | 251 (45.5%) |
|  |  | I/D | 116 (43%) | 253 (45.8%) |
|  |  | I/I | 26 (9.6%) | 48 (8.7%) |
|  | Shandong | D/D | 119 (46%) | 151 (46.6%) |
|  |  | I/D | 108 (41.7%) | 135 (41.7%) |
|  |  | I/I | 32 (12.4%) | 38 (11.7%) |

Table S3. Associations between *TYMS* polymorphisms and VSD in two independent case-control studies.

| SNP | Group | Genotype | Case | Control | P value* |
| --- | --- | --- | --- | --- | --- |
| rs58808873 | Shanghai | C/C | 161 (70.3%) | 373 (67.6%) |  |
|  |  | C/T | 59 (25.8%) | 166 (30.1%) | 0.48 |
|  |  | T/T | 9 (3.9%) | 13 (2.4%) |  |
|  | Shandong | C/C | 153 (70.2%) | 229 (70.7%) |  |
|  |  | C/T | 51 (23.4%) | 84 (25.9%) | 0.24 |
|  |  | T/T | 14 (6.4%) | 11 (3.4%) |  |
| rs9967368 | Shanghai | C/C | 79 (34.5%) | 168 (30.4%) |  |
|  |  | G/C | 107 (46.7%) | 280 (50.7%) | 0.63 |
|  |  | G/G | 43 (18.8%) | 104 (18.8%) |  |
|  | Shandong | C/C | 68 (31.2%) | 107 (33%) |  |
|  |  | G/C | 100 (45.9%) | 148 (45.7%) | 0.86 |
|  |  | G/G | 50 (22.9%) | 69 (21.3%) |  |
| rs56697663 | Shanghai | -/- | 90 (39.3%) | 201 (36.4%) |  |
|  |  | -/C | 101 (44.1%) | 268 (48.5%) | 0.99 |
|  |  | C/C | 38 (16.6%) | 83 (15%) |  |
|  | Shandong | -/- | 76 (34.9%) | 119 (36.7%) |  |
|  |  | -/C | 103 (47.2%) | 146 (45.1%) | 0.83 |
|  |  | C/C | 39 (17.9%) | 59 (18.2%) |  |
| rs2853741 | Shanghai | T/T | 65 (28.4%) | 132 (23.9%) |  |
|  |  | T/C | 116 (50.7%) | 290 (52.5%) | 0.54 |
|  |  | C/C | 48 (21%) | 130 (23.6%) |  |
|  | Shandong | T/T | 62 (28.4%) | 93 (28.7%) |  |
|  |  | T/C | 102 (46.8%) | 148 (45.7%) | 0.95 |
|  |  | C/C | 54 (24.8%) | 83 (25.6%) |  |
| rs2606241 | Shanghai | A/A | 88 (38.4%) | 236 (42.8%) |  |
|  |  | C/A | 112 (48.9%) | 250 (45.3%) | 0.15 |
|  |  | C/C | 29 (12.7%) | 66 (12%) |  |
|  | Shandong | A/A | 81 (37.2%) | 112 (34.6%) |  |
|  |  | C/A | 91 (41.7%) | 145 (44.8%) | 0.77 |
|  |  | C/C | 46 (21.1%) | 67 (20.7%) |  |
| rs9952504 | Shanghai | A/A | 197 (86%) | 480 (87%) |  |
|  |  | A/G | 30 (13.1%) | 67 (12.1%) | 0.35 |
|  |  | G/G | 2 (0.9%) | 5 (0.9%) |  |
|  | Shandong | A/A | 171 (78.4%) | 270 (83.3%) |  |
|  |  | A/G | 43 (19.7%) | 50 (15.4%) | 0.3 |
|  |  | G/G | 4 (1.8%) | 4 (1.2%) |  |
| rs34743033 | Shanghai | I/I | 149 (65.1%) | 349 (63.2%) |  |
|  |  | I/D | 70 (30.6%) | 184 (33.3%) | 0.42 |
|  |  | D/D | 10 (4.4%) | 19 (3.4%) |  |
|  | Shandong | I/I | 135 (61.9%) | 203 (62.6%) |  |
|  |  | I/D | 76 (34.9%) | 110 (34%) | 0.98 |
|  |  | D/D | 7 (3.2%) | 11 (3.4%) |  |
| rs73366471 | Shanghai | A/A | 195 (85.2%) | 483 (87.5%) |  |
|  |  | A/G | 31 (13.5%) | 65 (11.8%) | 0.34 |
|  |  | G/G | 3 (1.3%) | 4 (0.7%) |  |
|  | Shandong | A/A | 197 (90.4%) | 290 (89.5%) |  |
|  |  | A/G | 19 (8.7%) | 32 (9.9%) | 0.86 |
|  |  | G/G | 2 (0.9%) | 2 (0.6%) |  |
| rs699517 | Shanghai | T/T | 108 (47.2%) | 282 (51.1%) |  |
|  |  | C/T | 97 (42.4%) | 226 (40.9%) | 0.41 |
|  |  | C/C | 24 (10.5%) | 44 (8%) |  |
|  | Shandong | T/T | 112 (51.4%) | 143 (44.1%) |  |
|  |  | C/T | 82 (37.6%) | 139 (42.9%) | 0.24 |
|  |  | C/C | 24 (11%) | 42 (13%) |  |
| rs2790 | Shanghai | A/A | 98 (42.8%) | 196 (35.5%) |  |
|  |  | A/G | 99 (43.2%) | 282 (51.1%) | 0.3 |
|  |  | G/G | 32 (14%) | 74 (13.4%) |  |
|  | Shandong | A/A | 67 (30.7%) | 120 (37%) |  |
|  |  | A/G | 120 (55%) | 156 (48.1%) | 0.22 |
|  |  | G/G | 31 (14.2%) | 48 (14.8%) |  |
| rs34489327 | Shanghai | D/D | 106 (46.3%) | 251 (45.5%) |  |
|  |  | I/D | 100 (43.7%) | 253 (45.8%) | 0.81 |
|  |  | I/I | 23 (10%) | 48 (8.7%) |  |
|  | Shandong | D/D | 102 (46.8%) | 151 (46.6%) |  |
|  |  | I/D | 89 (40.8%) | 135 (41.7%) | 0.99 |
|  |  | I/I | 27 (12.4%) | 38 (11.7%) |  |

*Genotype frequencies in case and control participants were compared using χ^2^ test with 2 degrees of freedom (df).

Table S4. *TYMS* main haplotype (Frequence>0.01) analysis of Shanghai group

| No. | rs58808873 | rs9967368 | rs56697663 | rs2853741 | rs2606241 | rs9952504 | rs34743033 | rs73366471 | rs699517 | rs2790 | rs34489327 | Freq | OR (95% CI)* | P-value^$^ |
| --- | --- | --- | --- | --- | --- | --- | --- | --- | --- | --- | --- | --- | --- | --- |
| 1 | G | C | T | A | A | T | I | A | T | C | D | 0.1713 | 1.00 | --- |
| 2 | G | C | T | A | A | T | I | A | T | T | D | 0.1146 | 1.61 (0.98 - 2.66) | 0.06 |
| 3 | G | G | T | A | A | T | I | A | T | C | D | 0.0622 | 1.31 (0.71 - 2.41) | 0.39 |
| 4 | A | G | C | G | C | T | I | A | C | T | I | 0.0523 | 0.70 (0.34 - 1.45) | 0.34 |
| 5 | G | G | C | G | C | T | I | A | C | T | I | 0.0308 | 2.11 (1.00 - 4.46) | 0.05 |
| 6 | G | C | T | A | A | T | D | A | T | C | D | 0.0259 | 1.17 (0.47 - 2.95) | 0.73 |
| 7 | G | C | C | G | C | T | D | A | C | T | I | 0.0252 | 1.37 (0.60 - 3.12) | 0.46 |
| 8 | G | C | C | G | C | T | I | A | T | T | D | 0.0244 | 0.80 (0.27 - 2.41) | 0.69 |
| 9 | A | G | C | G | C | T | D | A | C | T | I | 0.0196 | 1.84 (0.72 - 4.69) | 0.20 |
| 10 | G | G | C | G | C | T | D | A | C | T | I | 0.0165 | 0.84 (0.26 - 2.74) | 0.77 |
| 11 | G | C | T | A | A | T | D | A | T | T | D | 0.0163 | 3.18 (1.05 - 9.60) | **0.04** |
| 12 | G | G | C | G | C | T | I | A | T | T | D | 0.0145 | 1.65 (0.54 - 5.03) | 0.38 |
| 13 | G | G | T | G | C | C | I | G | T | T | D | 0.014 | 1.81 (0.66 - 4.93) | 0.25 |
| 14 | G | C | T | A | A | T | I | A | C | T | I | 0.0136 | 3.28 (1.07 - 10.04) | **0.04** |
| 15 | A | G | C | G | C | T | I | A | T | C | D | 0.0135 | 1.36 (0.44 - 4.16) | 0.59 |
| 16 | G | C | C | G | A | T | I | A | C | T | I | 0.0134 | 0.49 (0.12 - 1.99) | 0.32 |
| 17 | G | C | C | G | A | T | I | A | T | T | D | 0.0133 | 0.39 (0.06 - 2.56) | 0.33 |
| 18 | G | C | T | A | C | T | I | A | T | C | D | 0.0127 | 2.45 (0.90 - 6.70) | 0.08 |
| 19 | G | G | C | G | A | T | D | A | C | T | I | 0.0125 | 0.32 (0.04 - 2.37) | 0.27 |
| 20 | G | G | T | A | A | T | I | A | T | T | D | 0.0103 | 1.31 (0.32 - 5.37) | 0.71 |

*Adjusted by age and gender; ^$^P value for difference in haplotypes distributions between case and control subjects.

Table S5. *TYMS* main haplotype (Frequence>0.01) analysis of Shandong group

| No. | rs58808873 | rs9967368 | rs56697663 | rs2853741 | rs2606241 | rs9952504 | rs34743033 | rs73366471 | rs699517 | rs2790 | rs34489327 | Freq | OR (95% CI)* | P-value^$^ |
| --- | --- | --- | --- | --- | --- | --- | --- | --- | --- | --- | --- | --- | --- | --- |
| 1 | G | C | T | A | A | T | I | A | T | C | D | 0.1861 | 1.00 | --- |
| 2 | G | C | T | A | A | T | I | A | T | T | D | 0.1072 | 0.89 (0.48 - 1.64) | 0.71 |
| 3 | G | G | T | A | A | T | I | A | T | C | D | 0.0685 | 1.61 (0.83 - 3.13) | 0.16 |
| 4 | A | G | C | G | C | T | I | A | C | T | I | 0.0649 | 1.78 (0.95 - 3.34) | 0.07 |
| 5 | G | G | C | G | C | T | D | A | C | T | I | 0.0484 | 1.26 (0.62 - 2.54) | 0.52 |
| 6 | A | G | C | G | C | T | D | A | C | T | I | 0.0338 | 0.71 (0.32 - 1.57) | 0.40 |
| 7 | G | C | C | G | C | T | I | A | T | T | D | 0.0316 | 1.66 (0.66 - 4.16) | 0.28 |
| 8 | G | C | C | G | C | T | D | A | C | T | I | 0.0309 | 1.19 (0.49 - 2.88) | 0.70 |
| 9 | G | G | C | G | C | T | I | A | C | T | I | 0.0301 | 0.43 (0.16 - 1.17) | 0.10 |
| 10 | G | C | C | G | C | T | I | A | C | T | I | 0.0181 | 0.51 (0.14 - 1.88) | 0.31 |
| 11 | G | C | T | A | A | C | I | A | T | C | D | 0.018 | 0.80 (0.16 - 4.07) | 0.79 |
| 12 | G | C | T | G | A | T | I | A | T | C | D | 0.0161 | 0.98 (0.31 - 3.08) | 0.97 |
| 13 | G | C | T | A | C | T | I | A | T | C | D | 0.0159 | 1.15 (0.37 - 3.52) | 0.81 |
| 14 | G | G | C | G | C | T | I | A | T | T | D | 0.0156 | 2.07 (0.57 - 7.51) | 0.27 |
| 15 | G | C | T | A | A | T | D | A | T | C | D | 0.0143 | 0.81 (0.20 - 3.30) | 0.76 |
| 16 | G | G | T | A | A | C | I | A | T | C | D | 0.0141 | 0.87 (0.26 - 2.96) | 0.83 |
| 17 | A | G | C | G | C | T | I | A | T | C | D | 0.0118 | 3.22 (0.77 - 13.47) | 0.11 |
| 18 | A | G | C | G | C | T | I | A | T | T | D | 0.0113 | 1.22 (0.21 - 7.10) | 0.83 |

*Adjusted by age and gender; ^$^P value for difference in haplotypes distributions between case and control subjects.

Table S6. High frequency haplotypes (Frequency > 5%) distribution in Shanghai and Shandong group.

| No. | rs58808873 | rs9967368 | rs56697663 | rs2853741 | rs2606241 | rs9952504 | rs34743033 | rs73366471 | rs699517 | rs2790 | rs34489327 | Haplotype Frequency in Control Samples | | | Haplotype Frequency in All Samples | | |
| --- | --- | --- | --- | --- | --- | --- | --- | --- | --- | --- | --- | --- | --- | --- | --- | --- | --- |
|  |  |  |  |  |  |  |  |  |  |  |  | Shanghai | Shandong | P-value* | Shanghai | Shandong | P-value* |
| 1 | G | C | T | A | A | T | I | A | T | C | D | 0.1846 | 0.1992 | (reference) | 0.1729 | 0.1838 | (reference) |
| 2 | G | C | T | A | A | T | I | A | T | T | D | 0.1125 | 0.1198 | 0.49 | 0.1155 | 0.1091 | 0.74 |
| 3 | G | G | T | A | A | T | I | A | T | C | D | 0.0631 | 0.0572 | 0.99 | 0.0622 | 0.0686 | 0.56 |
| 4 | A | G | C | G | C | T | I | A | C | T | I | 0.0634 | 0.0539 | 0.61 | 0.0515 | 0.0649 | 0.4 |

*P value for difference in haplotypes distributions between Shanghai and Shandong subjects.
