# Supplementary material for: Genetic Polymorphisms of the TYMS Gene Are Not Associated with Congenital Cardiac Septal Defects in a Han Chinese Population
Source: PLoS One. 2012 Feb 23;7(2):e31644. doi: 10.1371/journal.pone.0031644 (PMC3285645; doi:10.1371/journal.pone.0031644)
Supplement: Table S2 — The genotype frequency of the 11 identified TYMS polymorphisms in CCSDs patients and controls. (DOC) [file pone.0031644.s002.doc]

Table S2. The genotype frequency of the 11 identified *TYMS* polymorphisms in CCSDs patients and controls

| SNP | Group | Genotype | Case | Control |
| --- | --- | --- | --- | --- |
| rs58808873 | Shanghai | C/C | 185 (68.5%) | 373 (67.6%) |
|  |  | C/T | 76 (28.1%) | 166 (30.1%) |
|  |  | T/T | 9 (3.3%) | 13 (2.4%) |
|  | Shandong | C/C | 176 (68%) | 229 (70.7%) |
|  |  | C/T | 67 (25.9%) | 84 (25.9%) |
|  |  | T/T | 16 (6.2%) | 11 (3.4%) |
| rs9967368 | Shanghai | C/C | 88 (32.6%) | 168 (30.4%) |
|  |  | G/C | 131 (48.5%) | 280 (50.7%) |
|  |  | G/G | 51 (18.9%) | 104 (18.8%) |
|  | Shandong | C/C | 77 (29.7%) | 107 (33%) |
|  |  | G/C | 125 (48.3%) | 148 (45.7%) |
|  |  | G/G | 57 (22%) | 69 (21.3%) |
| rs56697663 | Shanghai | -/- | 101 (37.4%) | 201 (36.4%) |
|  |  | -/C | 127 (47%) | 268 (48.5%) |
|  |  | C/C | 42 (15.6%) | 83 (15%) |
|  | Shandong | -/- | 89 (34.4%) | 119 (36.7%) |
|  |  | -/C | 122 (47.1%) | 146 (45.1%) |
|  |  | C/C | 48 (18.5%) | 59 (18.2%) |
| rs2853741 | Shanghai | T/T | 73 (27%) | 132 (23.9%) |
|  |  | T/C | 145 (53.7%) | 290 (52.5%) |
|  |  | C/C | 52 (19.3%) | 130 (23.6%) |
|  | Shandong | T/T | 71 (27.4%) | 93 (28.7%) |
|  |  | T/C | 122 (47.1%) | 148 (45.7%) |
|  |  | C/C | 66 (25.5%) | 83 (25.6%) |
| rs2606241 | Shanghai | A/A | 101 (37.4%) | 236 (42.8%) |
|  |  | C/A | 138 (51.1%) | 250 (45.3%) |
|  |  | C/C | 31 (11.5%) | 66 (12%) |
|  | Shandong | A/A | 101 (39%) | 112 (34.6%) |
|  |  | C/A | 105 (40.5%) | 145 (44.8%) |
|  |  | C/C | 53 (20.5%) | 67 (20.7%) |
| rs9952504 | Shanghai | A/A | 232 (85.9%) | 480 (87%) |
|  |  | A/G | 35 (13%) | 67 (12.1%) |
|  |  | G/G | 3 (1.1%) | 5 (0.9%) |
|  | Shandong | A/A | 208 (80.3%) | 270 (83.3%) |
|  |  | A/G | 47 (18.1%) | 50 (15.4%) |
|  |  | G/G | 4 (1.5%) | 4 (1.2%) |
| rs34743033 | Shanghai | I/I | 174 (64.4%) | 349 (63.2%) |
|  |  | I/D | 83 (30.7%) | 184 (33.3%) |
|  |  | D/D | 13 (4.8%) | 19 (3.4%) |
|  | Shandong | I/I | 165 (63.7%) | 203 (62.6%) |
|  |  | I/D | 87 (33.6%) | 110 (34%) |
|  |  | D/D | 7 (2.7%) | 11 (3.4%) |
| rs73366471 | Shanghai | A/A | 233 (86.3%) | 483 (87.5%) |
|  |  | A/G | 34 (12.6%) | 65 (11.8%) |
|  |  | G/G | 3 (1.1%) | 4 (0.7%) |
|  | Shandong | A/A | 233 (90%) | 290 (89.5%) |
|  |  | A/G | 24 (9.3%) | 32 (9.9%) |
|  |  | G/G | 2 (0.8%) | 2 (0.6%) |
| rs699517 | Shanghai | T/T | 128 (47.4%) | 282 (51.1%) |
|  |  | C/T | 115 (42.6%) | 226 (40.9%) |
|  |  | C/C | 27 (10%) | 44 (8%) |
|  | Shandong | T/T | 131 (50.6%) | 143 (44.1%) |
|  |  | C/T | 100 (38.6%) | 139 (42.9%) |
|  |  | C/C | 28 (10.8%) | 42 (13%) |
| rs2790 | Shanghai | A/A | 110 (40.7%) | 196 (35.5%) |
|  |  | A/G | 121 (44.8%) | 282 (51.1%) |
|  |  | G/G | 39 (14.4%) | 74 (13.4%) |
|  | Shandong | A/A | 78 (30.1%) | 120 (37%) |
|  |  | A/G | 143 (55.2%) | 156 (48.1%) |
|  |  | G/G | 38 (14.7%) | 48 (14.8%) |
| rs34489327 | Shanghai | D/D | 128 (47.4%) | 251 (45.5%) |
|  |  | I/D | 116 (43%) | 253 (45.8%) |
|  |  | I/I | 26 (9.6%) | 48 (8.7%) |
|  | Shandong | D/D | 119 (46%) | 151 (46.6%) |
|  |  | I/D | 108 (41.7%) | 135 (41.7%) |
|  |  | I/I | 32 (12.4%) | 38 (11.7%) |
