# Supplementary material for: Genetic Polymorphisms of the TYMS Gene Are Not Associated with Congenital Cardiac Septal Defects in a Han Chinese Population
Source: PLoS One. 2012 Feb 23;7(2):e31644. doi: 10.1371/journal.pone.0031644 (PMC3285645; doi:10.1371/journal.pone.0031644)
Supplement: Table S3 — Associations between TYMS polymorphisms and VSD in two independent case-control studies. (DOC) [file pone.0031644.s003.doc]

Table S3. Associations between *TYMS* polymorphisms and VSD in two independent case-control studies.

| SNP | Group | Genotype | Case | Control | P value* |
| --- | --- | --- | --- | --- | --- |
| rs58808873 | Shanghai | C/C | 161 (70.3%) | 373 (67.6%) |  |
|  |  | C/T | 59 (25.8%) | 166 (30.1%) | 0.48 |
|  |  | T/T | 9 (3.9%) | 13 (2.4%) |  |
|  | Shandong | C/C | 153 (70.2%) | 229 (70.7%) |  |
|  |  | C/T | 51 (23.4%) | 84 (25.9%) | 0.24 |
|  |  | T/T | 14 (6.4%) | 11 (3.4%) |  |
| rs9967368 | Shanghai | C/C | 79 (34.5%) | 168 (30.4%) |  |
|  |  | G/C | 107 (46.7%) | 280 (50.7%) | 0.63 |
|  |  | G/G | 43 (18.8%) | 104 (18.8%) |  |
|  | Shandong | C/C | 68 (31.2%) | 107 (33%) |  |
|  |  | G/C | 100 (45.9%) | 148 (45.7%) | 0.86 |
|  |  | G/G | 50 (22.9%) | 69 (21.3%) |  |
| rs56697663 | Shanghai | -/- | 90 (39.3%) | 201 (36.4%) |  |
|  |  | -/C | 101 (44.1%) | 268 (48.5%) | 0.99 |
|  |  | C/C | 38 (16.6%) | 83 (15%) |  |
|  | Shandong | -/- | 76 (34.9%) | 119 (36.7%) |  |
|  |  | -/C | 103 (47.2%) | 146 (45.1%) | 0.83 |
|  |  | C/C | 39 (17.9%) | 59 (18.2%) |  |
| rs2853741 | Shanghai | T/T | 65 (28.4%) | 132 (23.9%) |  |
|  |  | T/C | 116 (50.7%) | 290 (52.5%) | 0.54 |
|  |  | C/C | 48 (21%) | 130 (23.6%) |  |
|  | Shandong | T/T | 62 (28.4%) | 93 (28.7%) |  |
|  |  | T/C | 102 (46.8%) | 148 (45.7%) | 0.95 |
|  |  | C/C | 54 (24.8%) | 83 (25.6%) |  |
| rs2606241 | Shanghai | A/A | 88 (38.4%) | 236 (42.8%) |  |
|  |  | C/A | 112 (48.9%) | 250 (45.3%) | 0.15 |
|  |  | C/C | 29 (12.7%) | 66 (12%) |  |
|  | Shandong | A/A | 81 (37.2%) | 112 (34.6%) |  |
|  |  | C/A | 91 (41.7%) | 145 (44.8%) | 0.77 |
|  |  | C/C | 46 (21.1%) | 67 (20.7%) |  |
| rs9952504 | Shanghai | A/A | 197 (86%) | 480 (87%) |  |
|  |  | A/G | 30 (13.1%) | 67 (12.1%) | 0.35 |
|  |  | G/G | 2 (0.9%) | 5 (0.9%) |  |
|  | Shandong | A/A | 171 (78.4%) | 270 (83.3%) |  |
|  |  | A/G | 43 (19.7%) | 50 (15.4%) | 0.3 |
|  |  | G/G | 4 (1.8%) | 4 (1.2%) |  |
| rs34743033 | Shanghai | I/I | 149 (65.1%) | 349 (63.2%) |  |
|  |  | I/D | 70 (30.6%) | 184 (33.3%) | 0.42 |
|  |  | D/D | 10 (4.4%) | 19 (3.4%) |  |
|  | Shandong | I/I | 135 (61.9%) | 203 (62.6%) |  |
|  |  | I/D | 76 (34.9%) | 110 (34%) | 0.98 |
|  |  | D/D | 7 (3.2%) | 11 (3.4%) |  |
| rs73366471 | Shanghai | A/A | 195 (85.2%) | 483 (87.5%) |  |
|  |  | A/G | 31 (13.5%) | 65 (11.8%) | 0.34 |
|  |  | G/G | 3 (1.3%) | 4 (0.7%) |  |
|  | Shandong | A/A | 197 (90.4%) | 290 (89.5%) |  |
|  |  | A/G | 19 (8.7%) | 32 (9.9%) | 0.86 |
|  |  | G/G | 2 (0.9%) | 2 (0.6%) |  |
| rs699517 | Shanghai | T/T | 108 (47.2%) | 282 (51.1%) |  |
|  |  | C/T | 97 (42.4%) | 226 (40.9%) | 0.41 |
|  |  | C/C | 24 (10.5%) | 44 (8%) |  |
|  | Shandong | T/T | 112 (51.4%) | 143 (44.1%) |  |
|  |  | C/T | 82 (37.6%) | 139 (42.9%) | 0.24 |
|  |  | C/C | 24 (11%) | 42 (13%) |  |
| rs2790 | Shanghai | A/A | 98 (42.8%) | 196 (35.5%) |  |
|  |  | A/G | 99 (43.2%) | 282 (51.1%) | 0.3 |
|  |  | G/G | 32 (14%) | 74 (13.4%) |  |
|  | Shandong | A/A | 67 (30.7%) | 120 (37%) |  |
|  |  | A/G | 120 (55%) | 156 (48.1%) | 0.22 |
|  |  | G/G | 31 (14.2%) | 48 (14.8%) |  |
| rs34489327 | Shanghai | D/D | 106 (46.3%) | 251 (45.5%) |  |
|  |  | I/D | 100 (43.7%) | 253 (45.8%) | 0.81 |
|  |  | I/I | 23 (10%) | 48 (8.7%) |  |
|  | Shandong | D/D | 102 (46.8%) | 151 (46.6%) |  |
|  |  | I/D | 89 (40.8%) | 135 (41.7%) | 0.99 |
|  |  | I/I | 27 (12.4%) | 38 (11.7%) |  |

*Genotype frequencies in case and control participants were compared using χ^2^ test with 2 degrees of freedom (df).
