# Supplementary material for: Genetic Polymorphisms of the TYMS Gene Are Not Associated with Congenital Cardiac Septal Defects in a Han Chinese Population
Source: PLoS One. 2012 Feb 23;7(2):e31644. doi: 10.1371/journal.pone.0031644 (PMC3285645; doi:10.1371/journal.pone.0031644)
Supplement: Table S4 — TYMS main haplotype (Frequence>0.01) analysis of Shanghai group. (DOC) [file pone.0031644.s004.doc]

Table S4. *TYMS* main haplotype (Frequence>0.01) analysis of Shanghai group

| No. | rs58808873 | rs9967368 | rs56697663 | rs2853741 | rs2606241 | rs9952504 | rs34743033 | rs73366471 | rs699517 | rs2790 | rs34489327 | Freq | OR (95% CI)* | P-value^$^ |
| --- | --- | --- | --- | --- | --- | --- | --- | --- | --- | --- | --- | --- | --- | --- |
| 1 | G | C | T | A | A | T | I | A | T | C | D | 0.1713 | 1.00 | --- |
| 2 | G | C | T | A | A | T | I | A | T | T | D | 0.1146 | 1.61 (0.98 - 2.66) | 0.06 |
| 3 | G | G | T | A | A | T | I | A | T | C | D | 0.0622 | 1.31 (0.71 - 2.41) | 0.39 |
| 4 | A | G | C | G | C | T | I | A | C | T | I | 0.0523 | 0.70 (0.34 - 1.45) | 0.34 |
| 5 | G | G | C | G | C | T | I | A | C | T | I | 0.0308 | 2.11 (1.00 - 4.46) | 0.05 |
| 6 | G | C | T | A | A | T | D | A | T | C | D | 0.0259 | 1.17 (0.47 - 2.95) | 0.73 |
| 7 | G | C | C | G | C | T | D | A | C | T | I | 0.0252 | 1.37 (0.60 - 3.12) | 0.46 |
| 8 | G | C | C | G | C | T | I | A | T | T | D | 0.0244 | 0.80 (0.27 - 2.41) | 0.69 |
| 9 | A | G | C | G | C | T | D | A | C | T | I | 0.0196 | 1.84 (0.72 - 4.69) | 0.20 |
| 10 | G | G | C | G | C | T | D | A | C | T | I | 0.0165 | 0.84 (0.26 - 2.74) | 0.77 |
| 11 | G | C | T | A | A | T | D | A | T | T | D | 0.0163 | 3.18 (1.05 - 9.60) | **0.04** |
| 12 | G | G | C | G | C | T | I | A | T | T | D | 0.0145 | 1.65 (0.54 - 5.03) | 0.38 |
| 13 | G | G | T | G | C | C | I | G | T | T | D | 0.014 | 1.81 (0.66 - 4.93) | 0.25 |
| 14 | G | C | T | A | A | T | I | A | C | T | I | 0.0136 | 3.28 (1.07 - 10.04) | **0.04** |
| 15 | A | G | C | G | C | T | I | A | T | C | D | 0.0135 | 1.36 (0.44 - 4.16) | 0.59 |
| 16 | G | C | C | G | A | T | I | A | C | T | I | 0.0134 | 0.49 (0.12 - 1.99) | 0.32 |
| 17 | G | C | C | G | A | T | I | A | T | T | D | 0.0133 | 0.39 (0.06 - 2.56) | 0.33 |
| 18 | G | C | T | A | C | T | I | A | T | C | D | 0.0127 | 2.45 (0.90 - 6.70) | 0.08 |
| 19 | G | G | C | G | A | T | D | A | C | T | I | 0.0125 | 0.32 (0.04 - 2.37) | 0.27 |
| 20 | G | G | T | A | A | T | I | A | T | T | D | 0.0103 | 1.31 (0.32 - 5.37) | 0.71 |

*Adjusted by age and gender; ^$^P value for difference in haplotypes distributions between case and control subjects.
