# Supplementary material for: Genetic Polymorphisms of the TYMS Gene Are Not Associated with Congenital Cardiac Septal Defects in a Han Chinese Population
Source: PLoS One. 2012 Feb 23;7(2):e31644. doi: 10.1371/journal.pone.0031644 (PMC3285645; doi:10.1371/journal.pone.0031644)
Supplement: Table S5 — TYMS main haplotype (Frequence>0.01) analysis of Shandong group. (DOC) [file pone.0031644.s005.doc]

Table S5. *TYMS* main haplotype (Frequence>0.01) analysis of Shandong group

| No. | rs58808873 | rs9967368 | rs56697663 | rs2853741 | rs2606241 | rs9952504 | rs34743033 | rs73366471 | rs699517 | rs2790 | rs34489327 | Freq | OR (95% CI)* | P-value^$^ |
| --- | --- | --- | --- | --- | --- | --- | --- | --- | --- | --- | --- | --- | --- | --- |
| 1 | G | C | T | A | A | T | I | A | T | C | D | 0.1861 | 1.00 | --- |
| 2 | G | C | T | A | A | T | I | A | T | T | D | 0.1072 | 0.89 (0.48 - 1.64) | 0.71 |
| 3 | G | G | T | A | A | T | I | A | T | C | D | 0.0685 | 1.61 (0.83 - 3.13) | 0.16 |
| 4 | A | G | C | G | C | T | I | A | C | T | I | 0.0649 | 1.78 (0.95 - 3.34) | 0.07 |
| 5 | G | G | C | G | C | T | D | A | C | T | I | 0.0484 | 1.26 (0.62 - 2.54) | 0.52 |
| 6 | A | G | C | G | C | T | D | A | C | T | I | 0.0338 | 0.71 (0.32 - 1.57) | 0.40 |
| 7 | G | C | C | G | C | T | I | A | T | T | D | 0.0316 | 1.66 (0.66 - 4.16) | 0.28 |
| 8 | G | C | C | G | C | T | D | A | C | T | I | 0.0309 | 1.19 (0.49 - 2.88) | 0.70 |
| 9 | G | G | C | G | C | T | I | A | C | T | I | 0.0301 | 0.43 (0.16 - 1.17) | 0.10 |
| 10 | G | C | C | G | C | T | I | A | C | T | I | 0.0181 | 0.51 (0.14 - 1.88) | 0.31 |
| 11 | G | C | T | A | A | C | I | A | T | C | D | 0.018 | 0.80 (0.16 - 4.07) | 0.79 |
| 12 | G | C | T | G | A | T | I | A | T | C | D | 0.0161 | 0.98 (0.31 - 3.08) | 0.97 |
| 13 | G | C | T | A | C | T | I | A | T | C | D | 0.0159 | 1.15 (0.37 - 3.52) | 0.81 |
| 14 | G | G | C | G | C | T | I | A | T | T | D | 0.0156 | 2.07 (0.57 - 7.51) | 0.27 |
| 15 | G | C | T | A | A | T | D | A | T | C | D | 0.0143 | 0.81 (0.20 - 3.30) | 0.76 |
| 16 | G | G | T | A | A | C | I | A | T | C | D | 0.0141 | 0.87 (0.26 - 2.96) | 0.83 |
| 17 | A | G | C | G | C | T | I | A | T | C | D | 0.0118 | 3.22 (0.77 - 13.47) | 0.11 |
| 18 | A | G | C | G | C | T | I | A | T | T | D | 0.0113 | 1.22 (0.21 - 7.10) | 0.83 |

*Adjusted by age and gender; ^$^P value for difference in haplotypes distributions between case and control subjects.
