# Supplementary material for: Genetic Polymorphisms of the TYMS Gene Are Not Associated with Congenital Cardiac Septal Defects in a Han Chinese Population
Source: PLoS One. 2012 Feb 23;7(2):e31644. doi: 10.1371/journal.pone.0031644 (PMC3285645; doi:10.1371/journal.pone.0031644)
Supplement: Table S6 — High frequency haplotypes (Frequency>5%) distribution in Shanghai and Shandong group. (DOC) [file pone.0031644.s006.doc]

Table S6. High frequency haplotypes (Frequency > 5%) distribution in Shanghai and Shandong group.

| No. | rs58808873 | rs9967368 | rs56697663 | rs2853741 | rs2606241 | rs9952504 | rs34743033 | rs73366471 | rs699517 | rs2790 | rs34489327 | Haplotype Frequency in Control Samples | | | Haplotype Frequency in All Samples | | |
| --- | --- | --- | --- | --- | --- | --- | --- | --- | --- | --- | --- | --- | --- | --- | --- | --- | --- |
|  |  |  |  |  |  |  |  |  |  |  |  | Shanghai | Shandong | P-value* | Shanghai | Shandong | P-value* |
| 1 | G | C | T | A | A | T | I | A | T | C | D | 0.1846 | 0.1992 | (reference) | 0.1729 | 0.1838 | (reference) |
| 2 | G | C | T | A | A | T | I | A | T | T | D | 0.1125 | 0.1198 | 0.49 | 0.1155 | 0.1091 | 0.74 |
| 3 | G | G | T | A | A | T | I | A | T | C | D | 0.0631 | 0.0572 | 0.99 | 0.0622 | 0.0686 | 0.56 |
| 4 | A | G | C | G | C | T | I | A | C | T | I | 0.0634 | 0.0539 | 0.61 | 0.0515 | 0.0649 | 0.4 |

*P value for difference in haplotypes distributions between Shanghai and Shandong subjects.
